# The Effect of XPD/ERCC2 Polymorphisms on Gastric Cancer Risk among Different Ethnicities: A Systematic Review and Meta-Analysis

**DOI:** 10.1371/journal.pone.0043431

**Published:** 2012-09-13

**Authors:** Huiping Xue, Yan Lu, Bing Lin, Jinxian Chen, Feng Tang, Gang Huang

**Affiliations:** 1 Division of Gastroenterology and Hepatology, Renji Hospital, Shanghai Jiaotong University School of Medicine, Shanghai Institution of Digestive Disease, Key Laboratory of Gastroenterology and Hepatology, Ministry of Health, Shanghai Jiaotong University, Shanghai, People's Republic of China; 2 Department of Medicine, International Peace Hospital for the Protection of Mother and Child Health, Shanghai Jiaotong University School of Medicine, Shanghai, People's Republic of China; 3 Division of Nutrition, Zhongshan Hospital, Fudan University School of Medicine, Shanghai, People's Republic of China; 4 Department of General Surgery, Renji Hospital, Shanghai Jiaotong University School of Medicine, Shanghai, People's Republic of China; 5 Division of Pathology, Huashan Hospital, Fudan University School of Medicine, Shanghai, People's Republic of China; 6 Department of Nuclear Medicine, Renji Hospital, Shanghai Jiaotong University School of Medicine, Shanghai, People's Republic of China; IPO, Inst Port Oncology, Portugal

## Abstract

**Background:**

Potential xeroderma pigmentosum group D (XPD), also called excision repair cross-complimentary group two (ERCC2), Lys751Gln and Asp312Asn polymorphisms have been implicated in gastric cancer risk among different ethnicities.

**Methods:**

We aimed to explore the effect of XPD Lys751Gln and Asp312Asn polymorphisms on the susceptibility to gastric cancer among different ethnicities through a systematic review and meta-analysis. Each initially included article was scored for quality appraisal. Desirable data were extracted and registered into databases. 13 studies were ultimately eligible for the meta-analysis of Lys751Gln polymorphism and 9 studies for the meta-analysis of Asp312Asn polymorphism. We adopted the most probably appropriate genetic model (recessive model) for both Lys751Gln and Asp312Asn polymorphisms. Potential sources of heterogeneity were sought out via subgroup and sensitivity analyses, and publication biases were estimated.

**Results:**

Statistically significant findings were apparently noted in Asians but not in Caucasians for both XPD Lys751Gln and XPD Asp312Asn polymorphisms. A statistically significant finding could be seen in noncardia-type gastric cancer for XPD Lys751Gln polymorphism. A statistically significant finding could also be seen in high quality subgroup, small-and-moderate sample size subgroup, articles published after 2007, or PCR-RFLP genotyping subgroup for XPD Asp312Asn polymorphism.

**Conclusions:**

Our meta-analysis indicates that XPD Gln751Gln (CC) genotype and Asn312Asn (AA) genotype may seem to be more susceptible to gastric cancer in Asian populations but not in Caucasian populations, suggesting that the two genotypes may be important biomarkers of gastric cancer susceptibility for Asian populations, the assumption that needs to be further confirmed in well-designed studies among different ethnicities. Gln751Gln (CC) genotype may also be associated with noncardia-type gastric cancer risk, which should also be confirmed among different ethnicities in the future.

## Introduction

Although worldwide gastric cancer incidence has decreased, its mortality still ranks second [1]. In China, gastric cancer even constitutes one of the most lethal malignancies [2]. As is widely known, infectious, dietary, environmental, and genetic factors are implicated in gastric carcinogenesis, but those exposed to risk factors who ultimately develop gastric cancer comprises a minor proportion [3], suggesting that host genetic susceptibility plays an important role in gastric cancer risk among different ethnicities. Such various susceptibilities could be explained, in part, by single nucleotide polymorphisms (SNPs) of susceptible genes among different ethnicities [4], [5]. Our previously published meta-analysis papers have provided additional evidence for such ethnically susceptible differences [5], [6].

It is widely acknowledged that DNA must remain stable to undertake its crucial physiological functions, but it is persistently vulnerable to various endogenous and/or exogenous damages and thus its probable mutations could accumulate and carcinogenesis may occur due to the damaged DNA. DNA repair system, however, plays a vital role in maintaining the functions of normal cells and genome integrity through the reversal of the damaged DNA [7]. Inherited functional polymorphisms or accumulated mutations of DNA repair genes may influence the host capacity to repair the damaged DNA and thus modulate cancer risk [8]. SNPs of common DNA repair genes have been identified [9] and demonstrated to be linked to sporadic carcinogenesis [10], [11].

Nucleotide excision repair (NER), one of the major DNA repair pathways in humans, is capable of removing helix-distorting base lesions produced by ultraviolet light (UV) and an array of chemical agents [12]. XPD is believed to participate in DNA unwinding during NER and transcription because it possesses single-strand DNA-dependent ATPase and 5′–3′ DNA helicase activities [13], [14]. XPD (ERCC2) gene, located at chromosome 19q13.3, comprises 23 exons and its polymorphisms are thought to engender structural alterations of NER pathway and influence cancer susceptibility. The most widely investigated XPD polymorphisms in associations with cancer susceptibility comprise a nonsynonymous A>C substitution in exon 23 causing a lysine (Lys) to glutamine (Gln) substitution in codon 751 (Lys751Gln, rs1052559), a nonsynonymous G>A substitution in exon 10 leading to an aspartic acid (Asp) to asparagine (Asn) substitution in codon 312 (Asp312Asn, rs1799793), and a synonymous C>A substitution in exon 6 while conserving the arginine (R) residue in codon 156 (Arg156Arg, rs238406) [15].

In 2005, Huang WY *et al.* published the first study involved in XPD Lys751Gln polymorphism in relation to gastric cancer risk [16]. Since then, researchers have reported associations of XPD Lys751Gln, Asp312Asn, and/or Arg156Arg with the susceptibility to gastric cancer among different ethnicities, but with mixed or conflicting results [17]–[30]. There is only one published article concerning Arg156Arg polymorphism in relation to gastric cancer risk [28]. To date, there have been three relevant published meta-analysis papers focusing on XPD polymorphisms [31]–[33]. Two articles were mainly concerned with overall cancer susceptibilities rather than gastric cancer susceptibility in depth [31], [32], thus providing less information on its association with gastric cancer risk. More importantly, those three meta-analyses [31]–[33] all failed to adopt the most likely appropriate genetic model, and thus the authentic values of those statistical results could be compromised.

Accordingly, the aim of our meta-analysis was to explore, using the most appropriate genetic model, the effect of XPD polymorphisms on gastric cancer risk among different ethnicities and to identify possible sources of heterogeneity among the eligible studies.

## Materials and Methods

### Search Strategy

A systematic literature search was performed for articles regarding XPD/ERCC2 SNPs associated with gastric cancer risk. The MEDLINE, EMBASE databases, Chinese National Knowledge Infrastructure (CNKI), Web of Science, and BIOSIS databases were used simultaneously with the combination of terms “XPD”, “ERCC2”, “DNA repair”, “NER”, “Lys751Gln”, “Asp312Asn”, or “Arg156Arg”; “gene”; “polymorphism”, “variant”, or “SNP”; and “gastric cancer”, “gastric carcinoma”, or “stomach cancer” up to December 2011. The search was performed without any restriction on language. The scope of computerized literature search was expanded according to the reference lists of retrieved articles. The relevant original articles were also sought manually.

### Study Selection

Studies concerning the association of XPD/ERCC2 SNPs (Lys751Gln, Asp312Asn, and/or Arg156Arg) with gastric cancer risk were included if the following conditions were met: (i) any study described the association of at least one of XPD/ERCC2 SNPs with gastric cancer; (ii) any study reported the numbers of both controls and gastric cancer cases; (iii) results were expressed as odds ratio (OR) with 95% confidence intervals (CI); and (iv) studies were case-control or nested case-control ones.

### Methodological Quality Appraisal

To identify high-quality studies, we mainly adopted predefined criteria for Quality Appraisal initially proposed by Thakkinstian *et al.* [34], adapted by Camargo *et al.* [35], and refined by Xue *et al.* [5]–[7]. The criteria (seen in Table S1 online) cover credibility of controls, representativeness of cases, consolidation of gastric cancer, genotyping examination, and association assessment. Methodological quality was independently assessed by two investigators (Lin B and Lu Y). Disagreements were resolved through discussion. Scores ranged from the lowest zero to the highest ten. Articles with the score lower than 6.5 were considered “low-or-moderate quality” ones, whereas those no lower than 6.5 were thought of as “high quality” ones.

### Data Extraction

The following data from each article were extracted: authors, year of publication, country, ethnicity of participants (categorized as Caucasians, Asians, etc.), study design, source of controls, number of controls and of cases, genotyping method, distribution of age and gender, Lauren's classification (intestinal, diffuse, or mixed), anatomical classification (cardia or non-cardia cancer), smoking habit, drinking habit and *Helicobacter Pylori* infection status.

The data were extracted and registered into two databases independently by two investigators (Lin B and Lu Y) who were blind to journal names, institutions or fund grants. Any discrepancy between these two investigators was resolved by the investigator (Xue H), who participated in the discussion with them and made an ultimate decision.

### Statistical Analysis

All statistical analyses were performed using STATA statistical software (Version 10.1, STATA Corp, College Station, TX). Two-sided Ps<0.05 were considered statistically significant. Hardy-Weinberg equilibrium (HWE) in controls was calculated again in our meta-analysis. The chi-square goodness of fit was used to test deviation from HWE (significant at the 0.05 level).

Odds ratios (OR) and 95% confidence intervals (95% CI) were used to assess the strength of associations between XPD/ERCC2 SNPs and gastric cancer risk. OR_1_, OR_2_, and OR_3_ were calculated for genotypes CC versus AA, CA versus AA, and CC versus CA for XPD Lys751Gln polymorphism; AA versus GG, GA versus GG, and AA versus GA for XPD Asp312Asn polymorphism; AA versus CC, CA versus CC, and AA versus CA for XPD Arg156Arg polymorphism, respectively. The pairwise differences were used to determine the most appropriate genetic model. If OR_1_ = OR_3_≠1 and OR_2_ = 1, a recessive model is suggested. If OR_1_ = OR_2_≠1 and OR_3_ = 1, a dominant model is implied. If OR_2_ = 1/OR_3_ ≠1 and OR_1_ = 1, a complete overdominant model is suggested. If OR_1_>OR_2_>1 and OR_1_>OR_3_>1, or OR_1_<OR_2_<1 and OR_1_<OR_3_<1, a codominant model is indicated [34]. Take XPD Lys751Gln polymorphism as an example to illustrate it. If a dominant model was indicated, the original grouping was collapsed and the new group of C carriers (CC plus CA) was compared with AA genotype; if a recessive model was suggested, CC was compared to the group of AA plus CA; if a complete overdominant model was implied, the group of CC plus AA was compared with CA; or if a codominant model was insinuated, CC was compared with CA and with AA, respectively.

The Q statistic was used to assess heterogeneity across studies included in the meta-analysis. I-squared (*I* ^2^) value, representing variation in OR attributable to heterogeneity was then used to quantify the degree of such between-study heterogeneity [36]. According to recently published Venice criteria [37], “*I* ^2^<25% represents no heterogeneity, *I* ^2^ = 25–50% represents moderate heterogeneity, *I* ^2^ = 50–75% represents large heterogeneity, and *I* ^2^>75% represents extreme heterogeneity”. Between-study variance Tau-squared (τ^2^) value was also used to evaluate between-study variance. A fixed-effects model, using Mantel–Haenszel (M–H) method, was employed to calculate the pooled ORs when homogeneity existed on the basis of Q-test p value no less than 0.1.By contrast, a random-effects model, using DerSimonian and Laird method (D+L), was utilized if there was heterogeneity based on Q-test p value less than 0.1. Even for the homogeneity among-studies, D+L method was also used. Meta-regression analyses and subgroup analyses were utilized to explore and control potential sources of heterogeneity across studies. The significance of pooled ORs was tested by Z test (P<0.05 was considered significant).

Sensitivity analysis was performed, in which the meta-analysis estimates were computed after every one study being omitted in each turn.

Finally, publication bias was assessed by performing funnel plots qualitatively, and estimated by Begg's and Egger's tests quantitatively.

## Results

### Literature Search and Study Selection

After comprehensive searching, a total of 140 articles in English and 7 in Chinese were retrieved. In our meta-analysis were initially included altogether 15 studies [16]–[30] which met the inclusion criteria. Those 15 studies were preliminarily appropriate to the meta-analysis of the associations with gastric cancer regarding XPD SNPs, among which 13 studies concerned XPD Lys751Gln polymorphism [16]–[26], [28], [29], 9 studies concerned XPD Asp312Asn polymorphism [17]–[21], [23], [27], [28], [30], and only 1 study concerned XPD Arg156Arg polymorphism [28]. 1 article [23] was indexed in both English and Chinese searching engines.

Traditionally speaking, any study that deviated from HWE should have been removed; however, Minelli C et al. recently pointed out that studies that appear to deviate from HWE should be investigated further rather than just excluded unless there are other grounds for doubting the quality of the study [38]. To date, it is still inconclusive whether studies deviated from HWE should be included or excluded in conducting meta-analysis. In our meta-analysis, 3 studies concerned XPD Lys751Gln polymorphism [16], [25], [29] were deviated from HWE, and 3 studies concerned XPD Asp312Asn polymorphism [17], [27], [30] were also deviated from HWE; however, considering that the numbers of participants in those studies were large and given that sensitivity analyses would be conducted, we finally remained those studies in our meta-analysis.

**Figure 1:**
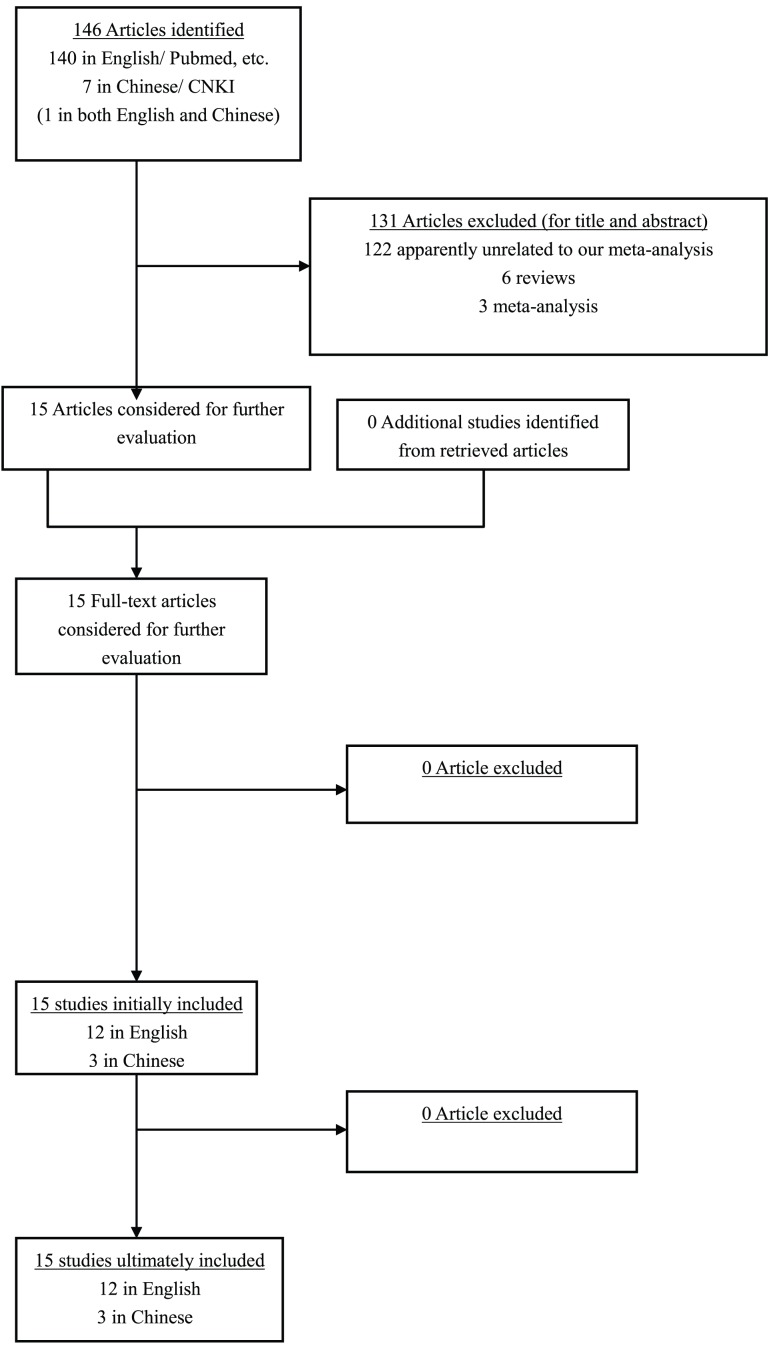
The flow chart of literature search and study selection.

Thus, 13 studies [16]–[26], [28], [29] with a total of 6344 controls and 2750 cases for XPD Lys751Gln polymorphism, and 9 studies [17]–[21], [23], [27], [28], [30] with 3429 controls and 1715 cases for XPD Asp312Asn polymorphism were ultimately eligible for the meta-analysis of XPD polymorphisms. The corresponding characteristics were seen in Table 1 and Table 2. The flow chart of literature search and study selection was illuminated in Figure 1.

**Table 1:** Study Characteristics of genotypes in gastric cancer cases and controls in the analysis of XPD Lys751Gln polymorphism.

First author	Year of publication	Quality assessment scores	Genotyping method	Total sample size	Number of controls	Number of cases	Study location	Ethnic group	P values for HWE	Controls, genotypes(n) Lys/Gln			All Cases, genotypes(n) Lys/Gln		
										Lys/Lys	Lys/Gln	Gln/Gln	Lys/Lys	Lys/Gln	Gln/Gln
Huang WY	2005	8.5	MALDI-TOF/hME	660	381	279	Poland	Caucasian	0.027863595	145	163	73	107	126	46
Ye W#	2006	8	PCR–RFLP	598	472	126	Sweden	Caucasian	0.114253032	198	203	71	49	61	16
Lou Y	2006	6	PCR–RFLP	438	200	238	China	Asian	0.377267735	164	33	3	205	30	3
Ruzzo A*	2007	7	PCR–RFLP	183	94	89	Italy	Caucasian	0.180617442	25	53	16	29	44	16
Zhou RM#	2007	7	PCR–RFLP	865	612	253	China	Asian	0.82380608	522	86	4	224	26	3
Capella G# ∧ ¶ ★	2008	9	Lightcycler	1417	1172	245	10 European countries	Caucasian	0.914544475	447	555	170	99	105	41
Doecke J#	2008	9	Sequenom iPLEX	1640	1337	303	Australia	Caucasian	0.220947678	575	588	174	127	140	36
Zhang CZ	2009	6.5	PCR–RFLP	419	212	207	China	Asian	0.439890159	172	39	1	166	39	2
Canbay E	2010	7	PCR–RFLP	287	247	40	Turkey	Turkish population□	0.922153834	102	114	31	14	18	8
Long XD∧ +	2010	7.5	TaqMan-PCR	977	616	361	China	Asian	6.33741E-08	400	164	52	139	151	71
Palli D	2010	8	TaqMan-PCR	841	546	295	Italy	Caucasian	0.098569581	177	284	85	90	157	48
Chen Z	2011	6.5	PCR–RFLP	547	339	208	China	Asian	0.698246164	282	55	2	166	40	2
Engin AB	2011	4	PCR–RFLP	222	116	106	Turkey	Turkish□ population	0.005846887	40	43	33	30	56	20

# **Data of cardia type of gastric cancer were accessible;**

∧ **Data of noncardia type of gastric cancer were accessible;**

* **Data of sporadic diffuse-type of gastric cancer were accessible;**

¶ **esophago-gastric junction adenocarcinoma (EGJAC) was treated as cardia type of gastric cancer in our meta-analysis.**

+ **Data of the status of** ***Helicobacter pylori*** **of gastric cancer were accessible.**

★ **Three kinds of controls (controls with severe chronic atrophic gastritis, controls without severe chronic atrophic gastritis, and total controls regardless of severe chronic atrophic gastritis) were presented in the study and total controls regardless of severe chronic atrophic gastritis were finally extracted into our database.**

□ **Here participants should be better considered as separate Turkish population conducted in our subgroup analysis due to their unknown ethnic backgrounds. RFLP: Restriction fragment length polymorphisms; TaqMan: TaqMan polymerase chain reaction method; MALDI-TOF/hME: SNPs were analyzed using Assisted Laser Desorption Ionization-Time of Flight mass spectrometry (MALDI-TOF MS) and homogeneous MassExtend (hME) chemistry (Sequenom Inc., San Diego, CA); Lightcycler: Polymorphisms were analysed in a LightCycler instrument by melting curve analysis of a fluorescently labelled sensor probe specific for each analysed variant, following manufacturer instructions (Roche Diagnostics, Mannheim, Germany), the results that were confirmed by a second genotyping method, such as restriction analysis, SSCP analysis or direct sequencing; Sequenom iPLEX : SNP typing was conducted using the Sequenom iPLEX protocol (Sequenom, San Diego, CA).**

**Table 2:** Study Characteristics of genotypes in gastric cancer cases and controls in the analysis of XPD Asp312Asn polymorphism.

First author	Year of publication	Quality assessment scores	Genotyping method	Total sample size	Number of controls	Number of cases	Study location	Ethnic group	P values for HWE	Controls, genotypes(n) G/A (Asp/Asn)			All Cases,genotypes(n) G/A (Asp/Asn)		
										Asp/Asp	Asp/Asn	Asn/Asn	Asp/Asp	Asp/Asn	Asn/Asn
Ye W#	2006	8	PCR–RFLP	596	470	126	Sweden	Caucasian	0.092544857	176	237	57	41	69	16
Lou Y	2006	6	PCR–RFLP	438	200	238	China	Asian	0.018951137	176	21	3	189	39	10
Ruzzo A*	2007	7	PCR–RFLP	210	121	89	Italy	Caucasian	0.061339561	41	67	13	23	46	20
Zhou RM# +	2007	7	PCR–RFLP	865	612	253	China	Asian	0.527426883	528	82	2	221	32	0
Capella G# ∧	2008	9	Lightcycler	1379	1135	244	10 European countries	Caucasian	0.985747613	444	532	159	110	96	38
Zhang CZ	2009	6.5	ARMS-PCR	419	212	207	China	Asian	0.636402675	132	72	8	75	117	15
Deng SL	2010	3.75	Direct sequencing	320	160	160	China	Asian	0.000155604	118	31	11	132	15	13
Chen Z	2011	6.5	PCR–RFLP	547	339	208	China	Asian	0.164678753	220	111	8	75	118	15
Yuan T	2011	6	Direct sequencing	370	180	190	China	Asian	9.72011E-05	133	35	12	156	18	16

# **Data of cardia type of gastric cancer were accessible;**

∧ **Data of noncardia type of gastric cancer were accessible;**

* **Data of sporadic diffuse-type of gastric cancer were accessible;**

+ **Data of the smoking habits of gastric cancer were accessible. RFLP: Restriction fragment length polymorphisms; Lightcycler: Polymorphisms were analysed in a LightCycler instrument by melting curve analysis of a fluorescently labelled sensor probe specific for each analysed variant, following manufacturer instructions (Roche Diagnostics, Mannheim, Germany), the results that were confirmed by a second genotyping method, such as restriction analysis, SSCP analysis or direct sequencing; ARMS: Amplification refractory mutation system.**

### Overall Meta-analysis, Meta-regression Analyses and Subgroup Analyses

For XPD Lys751Gln polymorphism, OR_1_ (p value), OR_2_ (p value), and OR_3_ (p value) were 1.22 (p = 0.266), 1.11 (p = 0.369), and 1.02 (p = 0.811), respectively, hardly insinuating a particular model effect of putative susceptible C allele. Heterogeneity chi-squared was 29.83 (d.f. = 12), p value was 0.003, and *I* ^2^ was 59.8%. After meta-regression analysis using single covariate (ethnicity composed of Caucasians, Asians, or Turkish population), p values of coefficient t value for Asians, Caucasians, or Turkish population were 0.001, 0.139, and 0.585, respectively; strongly indicating that Asians single covariate could mostly constitute the source of heterogeneity across studies. τ^2^ for Asians, Caucasians, or Turkish population single covariate were 0, 0.09178, and 0.1347, respectively. For Asians single covariate, τ^2^ decreased from 0.1233 to 0, indicating Asians single covariate could account for 100% of the source of heterogeneity across studies. When stratified by ethnicity subgroup analysis, OR_1_ (p value), OR_2_ (p value), and OR_3_ (p value) among Asian population were 2.63 (p = 0.002), 1.14 (p = 0.653), and 1.51 (p = 0.034), respectively, highly indicating a recessive model effect of putative susceptible C allele (OR_1_ = OR_3_≠1 and OR_2_  = 1).

For XPD Asp312Asn polymorphism, OR_1_ (p value), OR_2_ (p value), and OR_3_ (p value) were 1.75 (p = 0.015), 1.15 (p = 0.549), and 1.47 (p = 0.003), respectively, highly indicating a recessive model effect of putative susceptible A allele (OR_1_ = OR_3_≠1 and OR_2_ = 1). Heterogeneity chi-squared was 9.91 (d.f. = 8), p value was 0.272, and *I* ^2^ was 19.2%, indicating no heterogeneity across studies. After meta-regression analysis using single covariate (ethnicity composed of Caucasians or Asians), p value of coefficient t value for ethnicity single covariate was 0.277, indicating that ethnicity could constitute one of the sources of little heterogeneity across studies. τ^2^ decreased from 0.0354 to 0.02075, indicating ethnicity could account for 41.4% of the source of little heterogeneity across studies. Similarly, when stratified by ethnicity subgroup analysis, OR_1_ (p value), OR_2_ (p value), and OR_3_ (p value) among Asian population were 2.10 (p = 0.026), 1.22 (p = 0.574), and 1.80 (p = 0.008), respectively, further indicating a recessive model effect of putative susceptible A allele (OR_1_ = OR_3_≠1 and OR_2_ = 1).

Taken together, a recessive genetic model was ultimately chosen for both XPD Lys751Gln and XPD Asp312Asn polymorphisms in our meta-analysis.

**Figure 2:**
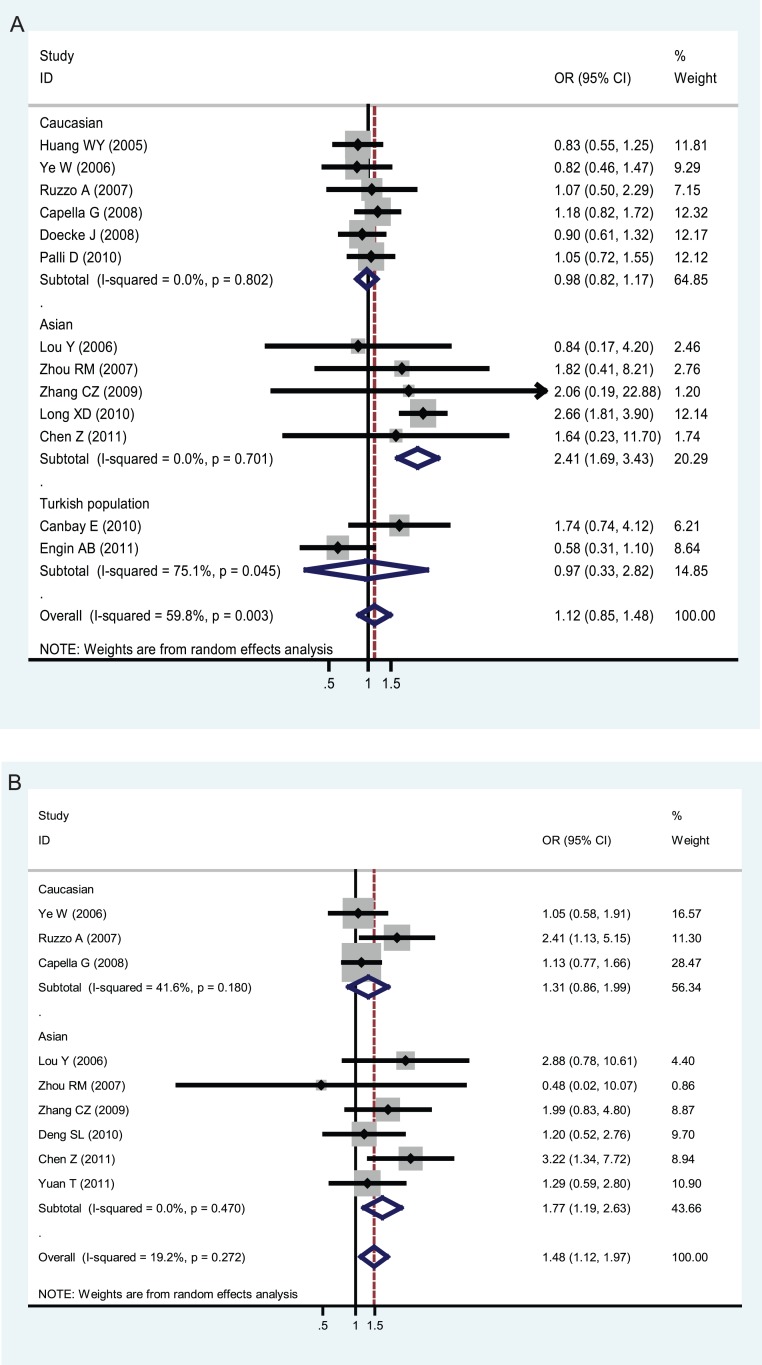
Odds ratios (ORs) for associations between XPD Lys751Gln and XPD Asp312Asn polymorphisms and gastric cancer risk (based on a recessive genetic model) among different ethnicities. in order of increasing publication year, 2005–2011. Studies were entered into the meta-analysis sequentially by year of publication. The sizes of the squares indicate the relative weight of each study. Weights were derived from random-effects analysis. Bars, 95% confidence interval (CI). A) The XPD Lys751Gln polymorphism in association with gastric cancer; B) XPD Asp312Asn polymorphism in association with gastric cancer.

In our meta-analysis, the available data were stratified, in the light of ethnic participants, into Caucasians, Asians, and Turkish population. In Figure 2, statistically significant findings were noted in Asians but not in Caucasians or Turkish population (both XPD Lys751Gln and XPD Asp312Asn polymorphisms). The pooled ORs (95% CIs, p value) were 2.41 (1.69–3.43, p = 0.000), 0.98 (0.82–1.17, p = 0.803), and 0.97 (0.33–2.82, p = 0.954) in Asians, Caucasians, and Turkish population (XPD Lys751Gln polymorphism in Part A) or 1.77 (1.19–2.63, p = 0.005) and 1.31 (0.86–1.99, p = 0.211) in Asians and Caucasians (XPD Asp312Asn polymorphism in Part B), respectively. As for XPD Lys751Gln polymorphism, P values of heterogeneity Q-statistic in Caucasians, Asians, and Turkish population were 0.802, 0.701, and 0.045; *I* ^2^ were 0.0%, 0.0%, and 75.1%; and τ^2^ were 0.0000, 0.0000, and 0.4489, respectively, demonstrating no heterogeneity within Caucasians or Asians, but extreme heterogeneity within Turkish population (shown in Table 3). As for XPD Asp312Asn polymorphism, P values of heterogeneity Q-statistic in Caucasians and Asians were 0.180 and 0.470; *I* ^2^ were 41.6% and 0.0%; andτ^2^ were 0.0587 and 0.0000, respectively, demonstrating no heterogeneity within Asians, but moderate heterogeneity within Caucasians (shown in Table 4).

**Table 3:** Stratification for the test of heterogeneity on XPD Lys751Gln based on a recessive model.

	Q-test			I^2^, %	τ^2^	OR(95%CI)	P value
	chi-squared	d.f.	p				
Overall	29.83	12	0.003	59.8	0.1233	1.12(0.85–1.48)	0.410
**Asians**	**2.19**	**4**	**0.701**	**0.0**	**0.0000**	**2.41 (1.69–3.43)**	**0.000**
Caucasians	2.33	5	0.802	0.0	0.0000	0.98 (0.82–1.17)	0.803
Turkish population	4.02	1	0.045	75.1	0.4489	0.97 (0.33–2.82)	0.954
Large sample	24.05	7	0.001	70.9	0.1437	1.17 (0.84–1.64)	0.351
Small-and-moderate sample	4.68	4	0.322	14.4	0.0409	0.96 (0.61–1.51)	0.858
High quality	25.08	10	0.005	60.1	0.1138	1.20 (0.91–1.60)	0.199
Low-and-moderate quality	0.17	1	0.684	0.0	0.0000	0.61 (0.34–1.11)	0.104
**High quality Asians**	**0.46**	**3**	**0.928**	**0.0**	**0.0000**	**2.54 (1.77–3.65)**	**0.000**
High quality Caucasians	2.33	5	0.802	0.0	0.0000	0.98 (0.82–1.17)	0803
Publication before or in 2007	1.27	4	0.866	0.0	0.0000	0.89 (0.66–1.19)	0.428
Publication after 2007	24.73	7	0.001	71.7	0.1805	1.23 (0.84–1.81)	0.285
**Non-cardia type**	**3.47**	**1**	**0.063**	**71.2**	**0.1155**	**2.03 (1.16–3.54)**	**0.013**
Cardia type	1.08	3	0.781	0.0	0.0000	0.88 (0.66–1.18)	0.403
PCR-RFLP genotyping	5.93	7	0.547	0.0	0.0000	0.94 (0.68–1.29)	0.701
TagMan PCR genotyping	11.04	1	0.001	90.9	0.3883	1.67 (0.68–4.14)	0.265

Only D+L ORs (95% CI) and P values of D+L estimates provided.

**Table 4:** Stratification for the test of heterogeneity on XPD Asp312Asn based on a recessive model.

	Q-test			I^2^, %	τ^2^	OR(95%CI)	P value
	chi-squared	d.f.	p				
**Overall**	**9.91**	**8**	**0.272**	**19.2**	**0.0354**	**1.48 (1.12–1.97)**	**0.007**
**Asians**	**4.58**	**5**	**0.470**	**0.0**	**0.0000**	**1.77 (1.19–2.63)**	**0.005**
Caucasians	3.43	2	0.180	41.6	0.0587	1.31 (0.86–1.99)	0.211
Large sample	5.42	3	0.144	44.6	0.1053	1.35 (0.82–2.21)	0.239
**Small-and-moderate sample**	**2.71**	**4**	**0.607**	**0.0**	**0.0000**	**1.74 (1.18–2.56)**	**0.005**
**High quality**	**8.55**	**5**	**0.128**	**41.5**	**0.0948**	**1.56 (1.05–2.33)**	**0.028**
Low-and-moderate quality	1.36	2	0.508	0.0	0.0000	1.42 (0.85–2.40)	0.184
**High quality Asians**	**1.68**	**2**	**0.433**	**0.0**	**0.0000**	**2.37 (1.29–4.36)**	**0.005**
High quality Caucasians	3.43	2	0.180	41.6	0.0587	1.31 (0.86–1.99)	0.211
Publication before or in 2007	4.34	3	0.227	30.9	0.1128	1.61(0.89–2.92)	0.115
**Publication after 2007**	**5.41**	**4**	**0.248**	**26.0**	**0.0452**	**1.46 (1.02–2.09)**	**0.041**
Cardia type	0.85	2	0.654	0.0	0.0000	0.89 (0.56–1.43)	0.642
**PCR-RFLP genotyping**	**6.59**	**4**	**0.159**	**39.3**	**0.1474**	**1.90 (1.09–3.31)**	**0.023**
Direct sequencing	0.02	1	0.902	0.0	0.0000	1.24 (0.70–2.20)	0.450

**Only D+L ORs (95% CI) and P values of D+L estimates provided.**

As shown in Table 3 and Table 4, specific data for XPD Lys751Gln and XPD Asp312Asn polymorphisms were stratified, respectively, on the basis of sample size, into two subgroups: large sample (the total number of controls and cases no less than 500) and small-and-moderate sample (the total number of controls and cases less than 500) subgroups. No statistically significant finding was noted in either small-and-moderate sample subgroup or large sample counterpart for XPD Lys751Gln polymorphism, given that the pooled ORs (95% CIs, p value) were 0.96 (0.61–1.51, p = 0.858) for the former and 1.17 (0.84–1.64, p = 0.351) for the latter, respectively; however a statistically significant finding was noted in small-and-moderate sample subgroup but not in large sample counterpart for Asp312Asn polymorphism, given that the pooled ORs (95% CIs, p value) were 1.74 (1.18–2.56, p = 0.005) for the former and 1.35 (0.82–2.21, p = 0.239) for the latter, respectively.

The data were also stratified, in accordance with the quality appraisal scores, into high quality (scores no less than 6.5) and low-and-moderate quality (scores less than 6.5) subgroups. As for XPD Lys751Gln polymorphism, no statistically significant finding was witnessed in either high quality subgroup or low-and-moderate quality counterpart, given that the pooled ORs (95% CIs, p value) were 1.20 (0.91–1.60, p = 0.199) for the former and 0.61 (0.34–1.11, p = 0.104) for the latter. When ethnicity sub-stratification was performed for high quality subgroup, a statistically significant finding was much apparently witnessed among Asians but no statistically significant finding was noted among Caucasians because the pooled ORs (95% CIs, p value) were 2.54 (1.77–3.65, p = 0.000) for the former and 0.98 (0.82–1.17, p = 0.803) for the latter, respectively. As for Asp312Asn polymorphism, a statistically significant finding was witnessed in high quality subgroup but not in low-and-moderate quality counterpart, given that the pooled ORs (95% CIs, p value) were 1.56 (1.05–2.33, p = 0.028) for the former and 1.42 (0.85–2.40, p = 0.184) for the latter. Likewise, when ethnicity sub-stratification was performed for high quality subgroup, a statistically significant finding was much apparently witnessed among Asians but no statistically significant finding was noted among Caucasians because the pooled ORs (95% CIs, p value) were 2.37 (1.29–4.36, p = 0.005) for the former and 1.31 (0.86–1.99, p = 0.211) for the latter, respectively.

The data were additionally stratified, in line with publication time, into the earlier publication subgroup (articles published before or in 2007) and the later publication subgroup (articles published after 2007). As for XPD Lys751Gln polymorphism, no statistically significant findings were observed on the grounds that the pooled ORs (95% CIs, p value) were 0.89 (0.66–1.19, p = 0.428) in the former and 1.23 (0.84–1.81, p = 0.285) in the latter, respectively. As for Asp312Asn polymorphism, a statistically significant finding was observed in the later publication subgroup but not in the earlier publication subgroup on the grounds that the pooled ORs (95% CIs, p value) were 1.46 (1.02–2.09, p = 0.041) in the former and 1.61(0.89–2.92, p = 0.115) in the latter, respectively.

When gastric cancer was classified into non-cardia (or distal) and cardia subtypes, a statistically significant finding was found among non-cardia type but not among cardia type for XPD Lys751Gln polymorphism on the grounds that the pooled ORs (95% CIs, p value) were 2.03 (1.16–3.54, p = 0.013) among non-cardia type and 0.88 (0.66–1.18, p = 0.403) among cardia type. As for Asp312Asn polymorphism, no statistically significant finding was observed among cardia type on the grounds that the pooled OR (95% CIs, p value) was 0.89 (0.56–1.43, p = 0.642), but OR (95% CIs, p value) regarding non-cardia type could not be calculated because only one study [21] clearly mentioned the numbers of genotypes of non-cardia type.

In terms of pathology, gastric cancer could be classified into intestinal, diffuse, or mixed subtypes, but only 1 study [19] clearly dealt with and mentioned the numbers of genotypes of diffuse subtype cancer; thus pathologic subtype stratification could not be done in our meta-analysis.

Confounding factors such as *Helicobacter pylori* infection or smoking status could not be analyzed in our meta-analysis because necessary relevant data of at least two studies could not be accessible in our meta-analysis.

And when genotyping techniques were considered, no statistically significant finding was noted in each genotyping technique subgroup for XPD Lys751Gln polymorphism because pooled ORs (95% CIs, p value) were 0.94 (0.68–1.29, p = 0.701) and 1.67 (0.68–4.14, p = 0.265) in RFLP and TagMan subgroups, respectively; however a statistically significant finding was noted in RFLP genotyping subgroup but not in Direct sequencing subgroup for Asp312Asn polymorphism because pooled ORs (95% CIs, p value) were 1.90 (1.09–3.31, p = 0.023) and 1.24 (0.70–2.20, p = 0.450), respectively.

### Sensitivity Analysis

**Figure 3:**
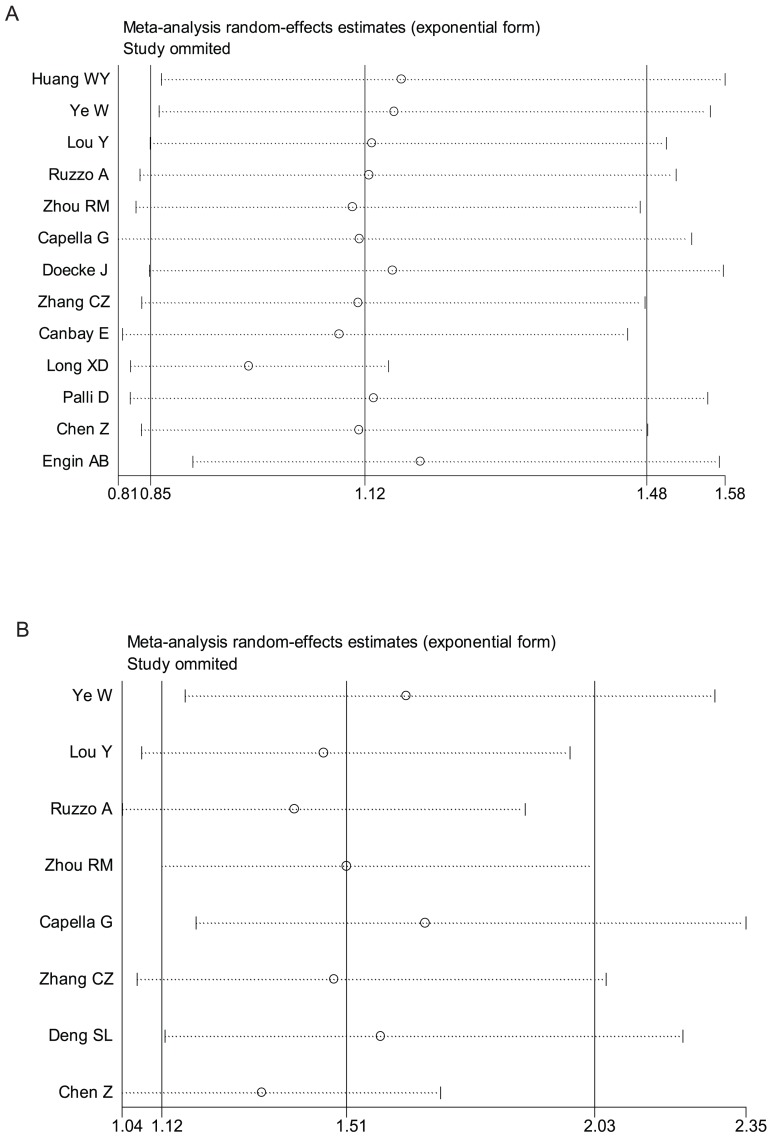
Influence analysis of the summary odds ratio coefficients on the association for XPD Lys751Gln and XPD Asp312Asn polymorphisms with gastric cancer risk (based on a recessive genetic model). Results were computed by omitting each study (on the left) in turn. Bars, 95% confidence interval. Meta-analysis random-effects estimates (exponential form) were used. A) Influence analysis of the summary odds ratio coefficients on the association for XPD Lys751Gln polymorphism with gastric cancer risk; B) Influence analysis of the summary odds ratio coefficients on the association for XPD Asp312Asn polymorphism with gastric cancer risk.

Meta-analyses were conducted repeatedly when each particular study had been removed. The results indicated that random-effects estimates before and after the deletion of each study were similar at large, suggesting moderately high stability of the meta-analysis results. For XPD Lys751Gln polymorphism, as shown in Figure 3 Part A, the most influencing single study on the overall pooled estimates seemed to be the study conducted by Long XD et al. [25], coincidently deviated from HWE, the sensitivity analysis, however, indicated moderately high stability of the results from the facts that the ORs (95% CI, p value) were 1.12(0.85–1.48, p = 0.410) before the removal of that study and 0.98 (0.83–1.15, p = 0.781) after the removal of that study. In view of the study [16] conducted by Huang WY et al. which is deviated from HWE, the ORs (95% CI, p value) were 1.12 (0.85–1.48, p = 0.410) before the removal of that study and 1.17 (0.87–1.58, p = 0.309) after the removal of that study for the all ethnicity, indicating high stability of the results. Similarly, after the removal of the study [29] conducted by Engin AB et al., also deviated from HWE, the OR (95% CI, p value) became 1.19 (0.91–1.57, p = 0.209) for the all ethnicity, indicating high stability of the results. If all the three deviated-HWE studies [16], [25], [29] were removed, the OR (95% CI, p value) became 1.06 (0.88–1.28, p = 0.555) for the all ethnicity, indicating moderately high stability of the results (The illustrating figures were omitted due to the length of paper).

For Asp312Asn polymorphism, as shown in Figure 3 Part B, the most influencing single study on the overall pooled estimates seemed to be the study conducted by Chen Z et al. [28], the sensitivity analysis, however, indicated moderately high stability of the results from the facts that the ORs (95% CI, p value) were 1.48 (1.12–1.97, p = 0.007) before the removal of that study and 1.32 (1.04–1.70, p = 0.025) after the removal of that study. If all the three deviated-HWE studies [17], [27], [30] were removed, the OR (95% CI, p value) became 1.56 (1.05–2.33, p = 0.028) for the all ethnicity, also indicating moderately high stability of the results (The illustrating figures were omitted due to the length of paper).

### Cumulative Meta-analysis

**Figure 4:**
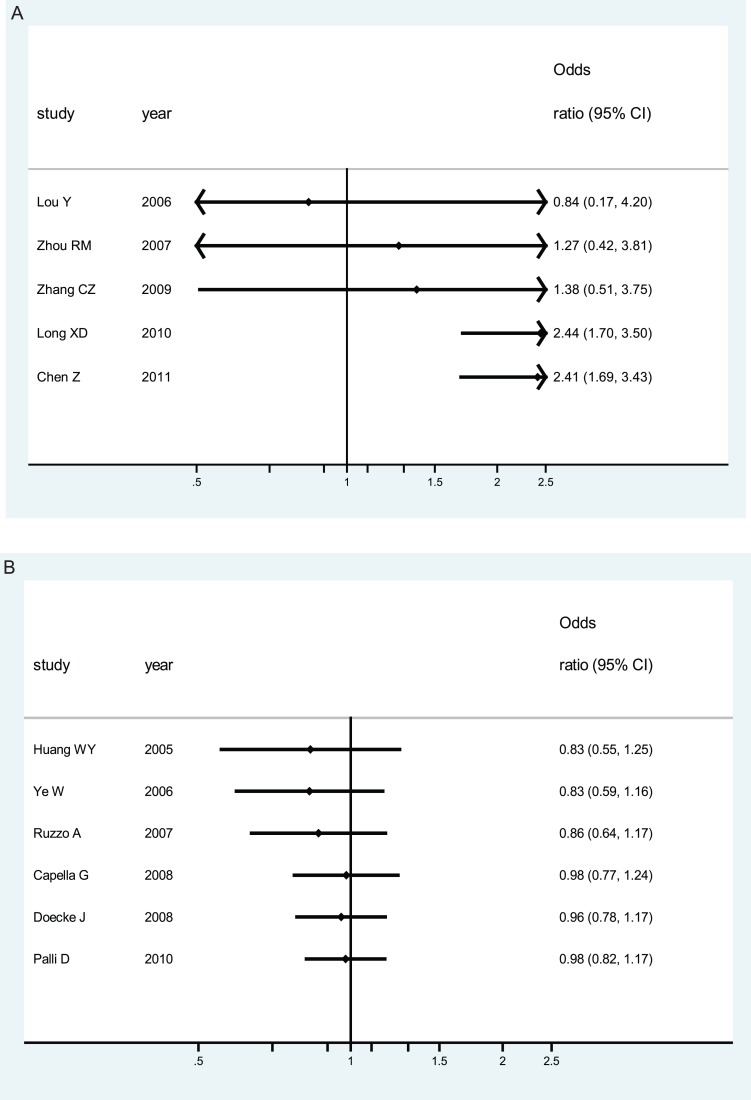
Cumulative meta-analysis of associations between XPD Lys751Gln polymorphism and gastric cancer risk among different ethnicities (based on a recessive genetic model). sorted by y publication time and total number of sample size; Horizontal line, the accumulation of estimates as each study was added rather than the estimate of a single study. A) among Asians; B) among Caucasians.

Cumulative meta-analyses of XPD Lys751Gln polymorphism association were also conducted among Asians and Caucasians via the assortment of publication time and total number of sample size. As shown in Figure 4 part A, the inclinations toward significant associations for XPD Lys751Gln polymorphism could be seen among Asians. In Figure 4 part B the inclinations toward null associations for XPD Lys751Gln polymorphism could be noted among Caucasians in chronological order. Similar inclinations could be observed for Asp312Asn polymorphism among different ethnicity populations (Figures not shown).

### Publication Bias Analysis

**Figure 5:**
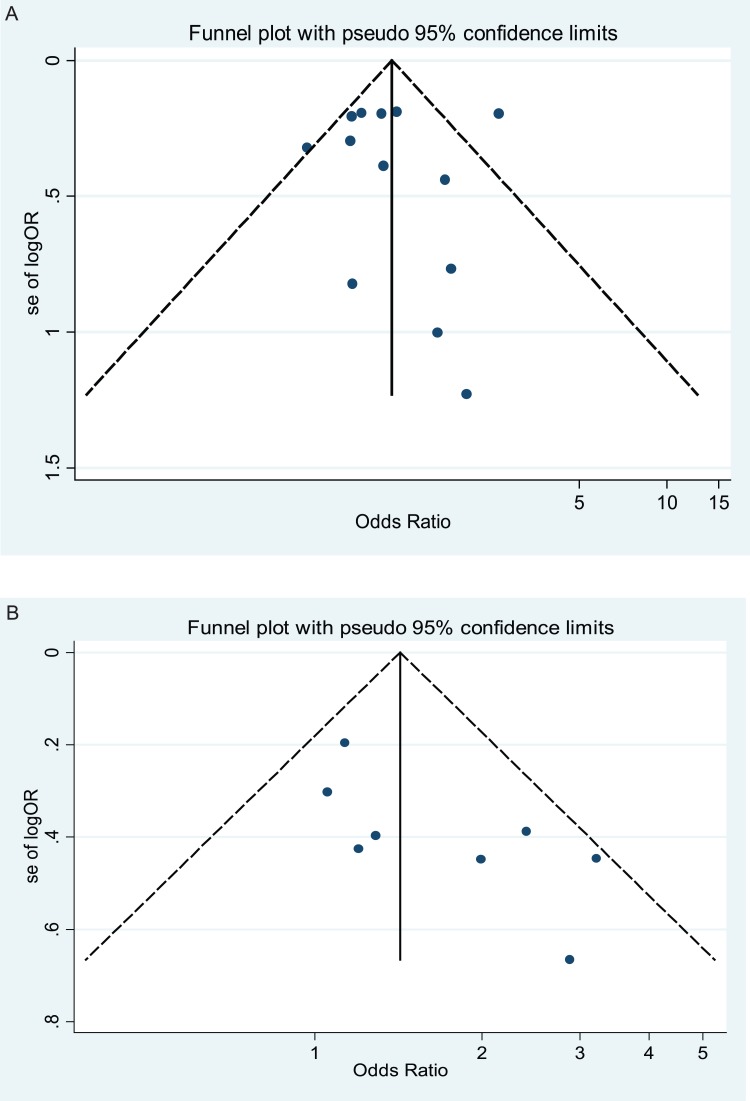
Funnel plot of publication bias for XPD Lys751Gln and XPD Asp312Asn polymorphisms with gastric cancer risk (based on a recessive genetic model). Note: Funnel plot with pseudo 95% confidence limits was used. A) Funnel plot of publication bias for XPD Lys751Gln polymorphism; B) Funnel plot of publication bias for XPD Asp312Asn polymorphism.

Publication bias was preliminarily examined by funnel plots qualitatively and estimated by Begg's and Egger's tests quantitatively. For XPD Lys751Gln polymorphism, its funnel plot (Figure 5A) showed that dots nearly symmetrically distributed, predominantly within pseudo 95% confidence limits. P values were 0.428 and 0.989 in Begg's test and Egger's test, respectively, insinuating no publication bias. For Asp312Asn polymorphism, its funnel plot (Figure 5B) showed that dots nearly symmetrically distributed, predominantly within pseudo 95% confidence limits. P values were 0.108 and 0.045 in Begg's test and Egger's test, respectively, insinuating no or a little publication bias.

## Discussion

In our meta-analysis statistically significant findings were apparently noted in Asians but not in Caucasians for both XPD Lys751Gln and XPD Asp312Asn polymorphisms. Also based on the findings of cumulative meta-analyses, the inclinations toward significant associations in Asians for both XPD Lys751Gln and XPD Asp312Asn polymorphisms could be obviously seen when sorted by publication time and total sample size. XPD Gln751Gln (CC) and Asn312Asn (AA) genotypes may seem to be more susceptible to gastric cancer in Asians. Our meta-analyses suggest that Gln751Gln (CC) and Asn312Asn (AA) genotypes may be important biomarkers of gastric cancer susceptibility for Asians, the assumption that needs to be further confirmed in future well-designed studies in Asian populations. Also the different or even conflicting risk associations, if so, among different ethnicities should be further meticulously investigated and reconfirmed in the future.

For XPD Asp312Asn SNP, although *I* ^2^ (%) was 19.2 and τ^2^ was 0.0354, as shown in Table 4, theoretically representing little or no heterogeneity; practically, after ethnicity subgroup analysis, the values of *I* ^2^ (%) and τ^2^ became 0.0 and 0.0000 for Asians and 41.6 and 0.0587 for Caucasians, respectively, further indicating that, actually, no heterogeneity existed among Asians studies rather than among Caucasians counterparts. Given this, we think that stratification or subgroup analyses are still needed in our meta-analysis. For XPD Asp312Asn polymorphism, our subgroup analyses also indicate that significant associations could be found in the small-and-moderate sample subgroup but not in the large sample counterpart. In large sample subgroup the ORs in the studies conducted by Capellá G et al. [21] and Ye W et al. [18] were both around 1.0, with the highest weight percentage to make the overall OR being statistically insignificant, whereas in small-and-moderate sample subgroup the influences of ORs in the studies conducted by Ruzzo A et al., Zhang CZ et al. and Lou Y et al. [19], [23], [17] were strong enough to make the overall OR to reach the significant value.

For XPD Asp312Asn polymorphism, a statistically significant finding was witnessed in high quality subgroup but not in low-and-moderate quality counterpart. When ethnicity sub-stratification was performed for such a high quality subgroup, a statistically significant finding was even more apparently witnessed among Asians but still no statistically significant finding was noted among Caucasians. For XPD Lys751Gln polymorphism, no statistically significant finding originally noted in high quality subgroup interestingly changed into a statistically significant finding among Asians but still not among Caucasians when ethnicity sub-stratification was performed for such a high quality subgroup. Those findings observed in different ethnic high quality subgroups further indicate the point that XPD Gln751Gln (CC) and Asn312Asn (AA) genotypes may be more susceptible to gastric cancer in Asians rather than in Caucasians. Certainly, high-quality studies should be more advocated to be designed in the future so as to accurately explore the real associations between XPD polymorphisms and gastric cancer risk among different ethnicities. Besides Asians and Caucasians, other ethnic participants, if possible, should be more appraised in the future.

Moreover, as for XPD Lys751Gln polymorphism, 4 [18], [20]–[22] out of 13 eligible studies were dealt with cardia gastric cancer and 2 [21], [25] with noncardia gastric cancer. A statistically significant finding could be noted with noncardia subgroup but not in cardia subgroup. Only 1 included study [19] in our meta-analysis mentioned pathologically sporadic diffuse-type gastric cancer and all the other studies did not mention pathologically Laurence's classification. As for XPD Asp312Asn polymorphism, 3 [18], [20], [21] out of 9 eligible studies were dealt with cardia gastric cancer and 1 [21] with noncardia gastric cancer. No statistically significant finding could be noted in cardia subgroup. Similarly, only 1 included study [19] mentioned pathologically sporadic diffuse-type gastric cancer and all the other studies did not mention pathologically Laurence's classification. As is widely known, cardia-type gastric cancer differs from noncardia-type gastric cancer in etiology, pathology, carcinogenesis, and/or prognosis [39]–[41], so is intestinal-type cancer versus diffuse-type cancer. It could be said that the indiscriminate combination of cardia-type and noncardia-type cases or intestinal-type and diffuse-type cases in the majority of eligible studies may mask or at least underestimate the real strength of the associations [5]–[7].

Furthermore, a variety of confounding factors such as *Helicobacter pylori* infection, alcoholic drinking, and smoking habits may be associated with increased damage to DNA repair [42]. Unfortunately, those factors could not be appraised in our meta-analysis due to the lack of relevant data.

With the advent of novel genotyping technologies like seminested polymerase chain reaction, TaqMan allelic discrimination test, or real-time PCR, we may witness an upsurge of genetic association studies in the future. As for XPD Asp312Asn polymorphism in our meta-analysis, a statistically significant finding could be noted in PCR-RFLP subgroup but not in Direct sequencing subgroup. As for XPD Lys751Gln polymorphism, however, no statistically significant finding was witnessed in either PCR-RFLP subgroup or TagMan PCR genotyping subgroup. Certainly, the difference should be concerned with extreme caution. Unfortunately, no direct sequencing was used among XPD Lys751Gln polymorphism studies. For a novel genotyping technique to be employed for the study of a particular genetic polymorphism, this technology, to our knowledge, should better be confirmed using direct sequencing. In that case, this new technology can be seen as valid as direct sequencing [43]. Or the sensitivity and specificity of those genotyping techniques need to be explored so as to seek out optimal approaches which could minimize the genotyping errors [5]–[7], [43]. And our opinion is that direct sequencing should be more used in future well-designed studies among different ethnicities.

Finally, the strength of our meta-analysis could be summarized as follows. We sought to find as many publications as we could by means of various searching approaches. The study that appeared to deviate from HWE was not excluded mechanically in our meta-analysis unless there are other convincing grounds for doubting the quality of the study [38]. Even so, we still performed the sensitivity analysis for the removal of all the deviated-HWE studies to further know the stability of the results, that is, the general impact of those deviated-HWE studies on the overall results. We laid more emphasis on assessing biases across studies and pinpointing the potential sources of heterogeneity via subgroup and sensitivity analyses. We comprehensively assessed the publication biases using several means like Begg's and Egger's tests as well as funnel plot tests. In view of this, we convince that the results of our meta-analysis, in essence, are sound and reliable.

To be sure, there are some unavoidable limitations in our meta-analysis. Firstly, the offered information from the included studies is inconsistent. Put it another way, the information about overall gastric cancer susceptibility is predominantly provided, while more important information about pathologic subtypes or anatomic subtypes of gastric cancer is less provided. Thus, the specific subtype results should be considered with caution. Secondly, with the merely published studies included in our meta-analysis, publication bias is very likely to occur, though no or a little statistically significant publication bias is indicated in our meta-analysis. Thirdly, moderate to severe heterogeneity could be witnessed among the included studies. So as to minimize the potential bias, we designed a rigorous protocol before conducting meta-analysis, and performed a scrupulous search for published studies using explicit methods for study selection, data extraction, statistical analysis, adoption of the most appropriate genetic model and sensitivity analysis.

In conclusion, XPD Gln751Gln (CC) and Asn312Asn (AA) genotypes may seem to be more susceptible to gastric cancer in Asian population but not in Caucasian population, suggesting that Gln751Gln (CC) and Asn312Asn (AA) genotypes may be important biomarkers of gastric cancer susceptibility for Asian population, the assumption that needs to be further confirmed in future well-designed studies among different ethnicities. Gln751Gln (CC) genotype may also be associated with the noncardia-type gastric cancer risk, the finding that also needs to be further confirmed.

## Supporting Information

Table S1 **Scales for Quality Assessment.**
(DOC)
