# Supplementary material for: The Effect of XPD/ERCC2 Polymorphisms on Gastric Cancer Risk among Different Ethnicities: A Systematic Review and Meta-Analysis
Source: PLoS One. 2012 Sep 13;7(9):e43431. doi: 10.1371/journal.pone.0043431 (PMC3441548; doi:10.1371/journal.pone.0043431)
Supplement: Table S1 — Scales for Quality Assessment. (DOC) [file pone.0043431.s001.doc]

**Table S1** - Scales for Quality Assessment

| **Criteria** | **Scores** |
| --- | --- |
| **Credibility of controls** |  |
| Population - or neighborhood -based | 2.5 |
| Blood donors | 2 |
| Healthy volunteers with description | 1.5 |
| Healthy volunteers without description | 1 |
| Nongastroenterology division hospital-based patients | 0.5 |
| Gastroenterology division hospital-based patients | 0.25 |
| Not described | 0 |
| **Representativeness of cases** |  |
| Selection from some population cancer registry | 2 |
| Selection from some gastroenterology or surgery service | 1.5 |
| Selection with broad inclusion or exclusion criteria | 0.5 |
| Selection without any description in detail | 0 |
| **Consolidation of gastric cancer** |  |
| Both histopathologic and anatomic confirmation | 2.5 |
| Only histopathologic or anatomic confirmation | 2 |
| Only by medical record | 1 |
| Not described | 0 |
| **Genotyping examination** |  |
| Under "blinded" condition | 1 |
| Lacking "blinded" condition or not mentioned | 0 |
| **Association assessment** |  |
| Assessed association between genotypes and gastric cancer with appropriate statistics and examining confounders and effect modifiers (including anatomic location of cancer, and histology) | 0.5^*^2=1.0 |
|  |  |
